# Calculation of the Half‐Life for the TL Signal of BeO Under Gamma and Alfa/Gamma Irradiation

**DOI:** 10.1002/bio.70251

**Published:** 2025-07-09

**Authors:** Rodrigo Martinez‐Baltezar, Carlos M. Martínez‐Martínez, L. Alberto Medina‐Juárez, Gustavo Sánchez‐Santacruz, Juan Azorín‐Nieto

**Affiliations:** ^1^ Departamento de Física Universidad Autónoma Metropolitana‐Iztapalapa Ciudad de México México

**Keywords:** charge trapping dynamics, half‐life, solid state dosimetry, Thermalox 995, trap stability

## Abstract

The thermoluminescent (TL) properties of BeO ceramics (Thermalox 995) were investigated under gamma and alpha/gamma irradiation. Glow curves in both cases exhibit three main peaks at 348 ± 1 K, 477 ± 2 K, and 616 ± 1 K. Activation energies for gammainduced peaks are 0.76 ± 0.03 eV, 1.18 ± 0.04 eV, and 1.11 ± 0.02 eV; these increase slightly under alpha/gamma irradiation to 0.81 ± 0.02 eV, 1.22 ± 0.03 eV, and 1.18 ± 0.01 eV. The kinetic orders are close to first order, with slightly higher values observed for the 477 K peak. Based on these parameters, the half‐lives of the TL components were calculated. For gamma: 131 s, 1.92 years, and 522 years; and for alpha/gamma: 168 s, 5.07 years, and 2067 years. Fading experiments on five samples showed deviations of less than 10% from theoretical values for the first peak. The half‐life also varied with irradiation time: the 477 K peak showed a decreasing trend, while the 616 K peak increased, indicating differences in trap‐filling mechanisms. Deconvolution analysis revealed that the second peak (around 477 K) exhibits the most linear dose response under both irradiation types. These results underscore the significance of analyzing half‐life behavior to understand the stability and performance of BeO as a dosimetric material under varied irradiation conditions.

## Graphical abstract

Thermoluminescent glow peaks in BeO Thermalox 995 exhibit irradiation‐type‐dependent half‐lives and activation energies, suggesting a complex dynamic trap behavior. The 478 K peak shows a linear dose response and distinct stability trends, emphasizing the importance of half‐life analysis in dosimetric applications under gamma and alpha/gamma irradiation.

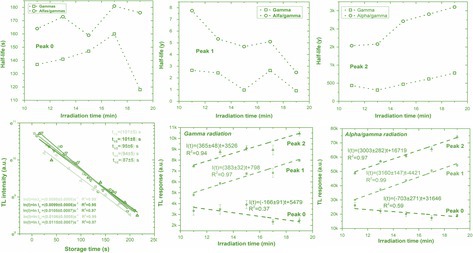

## 1 Introduction

Over the past few decades, the dosimetric properties of beryllium oxide (BeO) have been extensively studied by several research groups for applications in TL dosimetry (TLD) [1, 2, 3] and optically stimulated luminescence (OSL) dosimetry [4, 5]. BeO has attracted significant interest due to its excellent physical properties, including high thermal conductivity, high electrical resistivity, and strong resistance to thermal shock. These characteristics have led to widespread use as an insulating heat sink in the electronics industry [6]. Additionally, its high melting point (2570°C) and thermal stability make it suitable for use as a refractory material [7] and as a moderator in nuclear reactors [8].

Beyond its physical robustness, BeO is considered a tissue‐equivalent material with an effective atomic number of 7.13. It also possesses a wide bandgap (10.6 eV) and high thermal conductivity, which ensures rapid and uniform heat transfer during the readout process [9]. Furthermore, BeO is chemically stable, moldable, and resistant to many chemical agents, making it an attractive candidate for TLD applications. Nonetheless, its practical use is limited by certain drawbacks, such as the high toxicity of its powdered form, its marked sensitivity to light [10], the supralinear behavior of both TL and OSL dose–response curves [3, 11], and the strong phototransference observed in TL and OSL signals [12].

Some research groups have proposed methods to reduce these issues. Martinez‐Baltezar and Azorín‐Nieto [3] analyzed the TL glow curve of BeO over a wide dose range using two deconvolution methods: Glow‐Fit and “tgcd” in R. They found that the TL glow curve was composed of two TL peaks at approximately 477 and 586 K, both with activation energies of about 1.1 eV, with both methods confirming first‐order kinetics. The first TL peak shows a quadratic (supralinear) dose response, while the second peak exhibits a linear response, making it suitable for dosimetry applications. The study emphasizes the need to specify the deconvolution method when reporting TL responses [3].

Polymeris et al. [13] investigated the dose response behavior of BeO using both TL and continuous wave OSL (CW‐OSL) under various preheating and measurement temperatures. The study addresses concerns about supralinear dose responses and the role of thermal quenching, finding that strong supralinearity is observed in multiple TL peaks and OSL components. Reconstruction of signals (to correct for thermal quenching) significantly enhances this supralinearity. The optimal configuration for BeO dosimetry was found to be preheated at 220°C and performing OSL measurements at approximately 100°C, yielding a more linear response and reducing transfer effects [13].

Kharfi et al. [14] investigated the impact of combining different luminescence stimulation and reading modes on the TL intensity and glow curve behavior in BeO dosimeters, using the same X‐ray dose. Signal intensity and glow curve shape are significantly influenced by the stimulation order. Protocol 1 (IRSL → TSL) provides the best TL enhancement and allows multiple useful signals from the same irradiation. The optimal combination can improve dosimetric accuracy and efficiency.

Other research groups have investigated TL parameters and half‐life. Sasaki et al. [15] analyzed the TL properties and glow curve components of high‐purity BeO ceramic plates (Thermalox 995, > 99.5% BeO) using extremely slow heating rates. After irradiation with 5 Gy of 6 MV X‐rays, TL glow curves were measured with heating rates as slow as 0.005°C/s. General order kinetics (GOK) were applied for analysis, accounting for charge carrier recapture. Post‐annealing was performed between 50°C and 350°C, and component analysis revealed an additional low‐intensity TL component near the two main peaks. The activation energy and frequency factors for the low‐temperature peak were 1.15 eV and 1.11 × 10^11^ s^−1^, and for the high‐temperature peak, they were 1.74 eV and 8.65 × 10^13^ s^−1^. The study shows that extremely slow heating enhances TL efficiency, likely due to the activation of previously TL‐inactive charge carriers. On the other hand, Algarve and Caldas [2] investigated the TL properties of Thermalox 995, finding that using a linear heating rate of 5 K/s, two TL peaks were observed at approximately 475 and 621 K. The position of the second peak differs by 35 K from the value reported by Martinez‐Baltezar and Azorín‐Nieto [3] and Azorín‐Nieto et al. [16].

In a recent study, Azorín‐Nieto et al. [16] investigated the half‐life of the TL peak of Thermalox 995, finding that the half‐life of this material depends on the heating rate and storage temperature. They found that when using a heating rate of 5°C/s and a storage temperature of 20°C, the half‐life for the first and second peaks were 181.5 and 5.6 × 10^6^ days, respectively [16].

This paper reports the results of the calculations of the half‐life of each TL peak observed in the glow curve of Thermalox 995 as a function of gamma and alpha/gamma irradiation time. The deconvolution method was applied to determine the number and position of TL peaks, finding that the glow curve for Thermalox 995 is composed of three TL peaks at 348, 478, and 618 K for both types of radiation. The results suggest that the half‐life for this material depends on both types of radiation and irradiation time. To evaluate the uncertainty in the half‐life calculations, fading experiments were conducted. Furthermore, another finding of this research is that deconvolution methods can improve radiation measurements by studying individual TL components.

## 2 Materials and Methods

### 2.1 Thermoluminescence

The materials under investigation were BeO ceramic discs (Thermalox 995, Brush Beryllium Co., Elmore, OH, USA), with dimensions of 4 mm in diameter and 0.8 mm in thickness. According to the literature [17], the impurity content in BeO ceramics does not exceed 0.5%, with the following elemental concentrations: silicon (2150 ppm), magnesium (945 ppm), iron (100 ppm), calcium (61 ppm), aluminum (54 ppm), chromium (10 ppm), titanium (4 ppm), and copper (3 ppm).

To ensure the removal of any residual signal from previous exposures, the sample was annealed at 600°C for 30 min before irradiation. Irradiation with gamma and mixed alpha/gamma radiation was carried out using an Am‐241 radioactive source (1.11 GBq). This source emits alpha particles at a constant dose rate of 0.90 mGy/s and gamma rays at a constant dose rate of 3.81 μGy/s. Exposure times of 11, 13, 15, 17, and 19 min were employed for both radiation types. For gamma exposures, alpha particles were effectively blocked by interposing a 1 mm thick gold (Au) foil in front of the source. In all irradiation conditions, the sample was positioned at a fixed distance of 5 mm from the Am‐241 source. TL measurements were performed using a Harshaw 3500 TL analyzer (Thermo Scientific, USA) in the temperature range of 30°C–390°C, employing a linear heating rate of 5°C·s^−1^. All readings were conducted under a continuous nitrogen flow to minimize thermal noise generated by the heating planchet. A single sample was used for all experiments, and each experiment was repeated five times to ensure repeatability.

To determine both the kinetic parameters and the TL irradiation time‐response behavior for each individual glow peak, the deconvolution method based on the GOK model was employed [18]. The half‐life of isolated TL peaks as a function of gamma and gamma/alpha irradiation time was subsequently evaluated using the parameters obtained from the deconvolution analysis.

To evaluate the discrepancy between theoretical calculations and experimental measurements, five samples were irradiated for 10 min with mixed alpha/gamma radiation and subsequently stored at room temperature for various time intervals. This fading experiment enabled the determination of the half‐life of the 348 K TL peak.

### 2.2 Deconvolution Details

The “tgcd” is an open‐source R package developed by Peng et al. [18] and improved by Peng et al. [19]. This package utilizes some expressions from the GOK model, semi‐analytical expressions from the one‐trap, one‐recombination‐center model (OTOR), and the modified Levenberg–Marquardt algorithm to deconvolute TL curves.

For determining TL kinetics parameters, the GOK model (referred as g1 in the “tgcd” package) was employed, given that it is the most used model by researchers in this field. The transformed GOK equation for TL intensity is [20]

(1) $$\begin{array}{c} I=I_{M}b^{\frac{b}{b-1}}\exp\left(\frac{E}{\mathrm{kT}}\frac{T-T_{M}}{T_{M}}\right)\\ {\left[z_{M}+\left(b-1\right)\left(1-\Delta \right)\frac{T^{2}}{T_{M}^{2}}\exp\left(\frac{E}{\mathrm{kT}}\frac{T-T_{M}}{T_{M}}\right)\right]}^{\frac{b}{1-b}}, \end{array}$$

where

(2) $$z_{M}=1+\Delta_{M}\left(b-1\right),$$

(3) $$\Delta =\frac{2\mathrm{kT}}{E},$$

(4) $$\Delta_{M}=\frac{2kT_{M}}{E}.$$

Readers interested in more details of this derivation must consult [19, 20, 21].

The “tgcd” package includes second derivative calculations to assist users in determining the number and position of peaks in the glow curve. The quality of fit is measured using the figure of merit (FOM) value as follows [19]:

(5) $$\mathrm{FOM}=\sum_{i=1}^{i=n}\frac{\left|y_{i}-\hat{y_{i}}\right|}{A}\times 100,$$

where *y*_*i*_ is the *i*‐th observed value, $\hat{y_{i}}$ is the *i*‐th fitted value, *A* is the total area of the fit glow curve, and *n* is the number of fit data points [19].

### 2.3 Half‐Life Calculations

There are two ways to define the half‐life of TL traps [16, 22]. The first definition is the time required for the TL intensity I(t), of an isolated TL peak, to decrease to half of its initial value ($\tau_{1/2}$). The second definition is the time required for the total charge carriers trapped in a single trap n(t) to decay to half of its initial value ($t_{1/2}$). For obtaining an expression for the second definition, the GOK equation becomes as follows:

(6) $$\frac{\mathrm{dn}}{n^{b}}=-s^{\prime \prime }\exp\left(-\frac{E}{kT_{s}}\right)\mathrm{dt},$$

where *T*s is the storage temperature.

After integration, this equation becomes

(7) $$t_{1/2}=\left(\frac{1}{2^{1-b}}-1\right)\frac{n_{0}^{1-b}\exp\left(\frac{E}{kT_{s}}\right)}{s^{\prime \prime }\left(b-1\right)}.$$

This expression transforms into [16, 21, 22]

(8) $$t_{1/2}=\left(1-\frac{1}{2^{1-b}}\right)\left(\frac{kT_{m}^{2}}{\mathrm{qE}}\right)\frac{\exp\left[\frac{E}{k}\left(\frac{1}{T_{s}}-\frac{1}{T_{m}}\right)\right]}{\left(1-b\right)}\left(1+\frac{2kT_{m}\left(b-1\right)}{E}\right),$$

this equation enables the determination of the half‐life when *b*, *T*_m_, *T*_s_, and *E* are known parameters.

## 3 Results and Discussion

**FIGURE 1:**
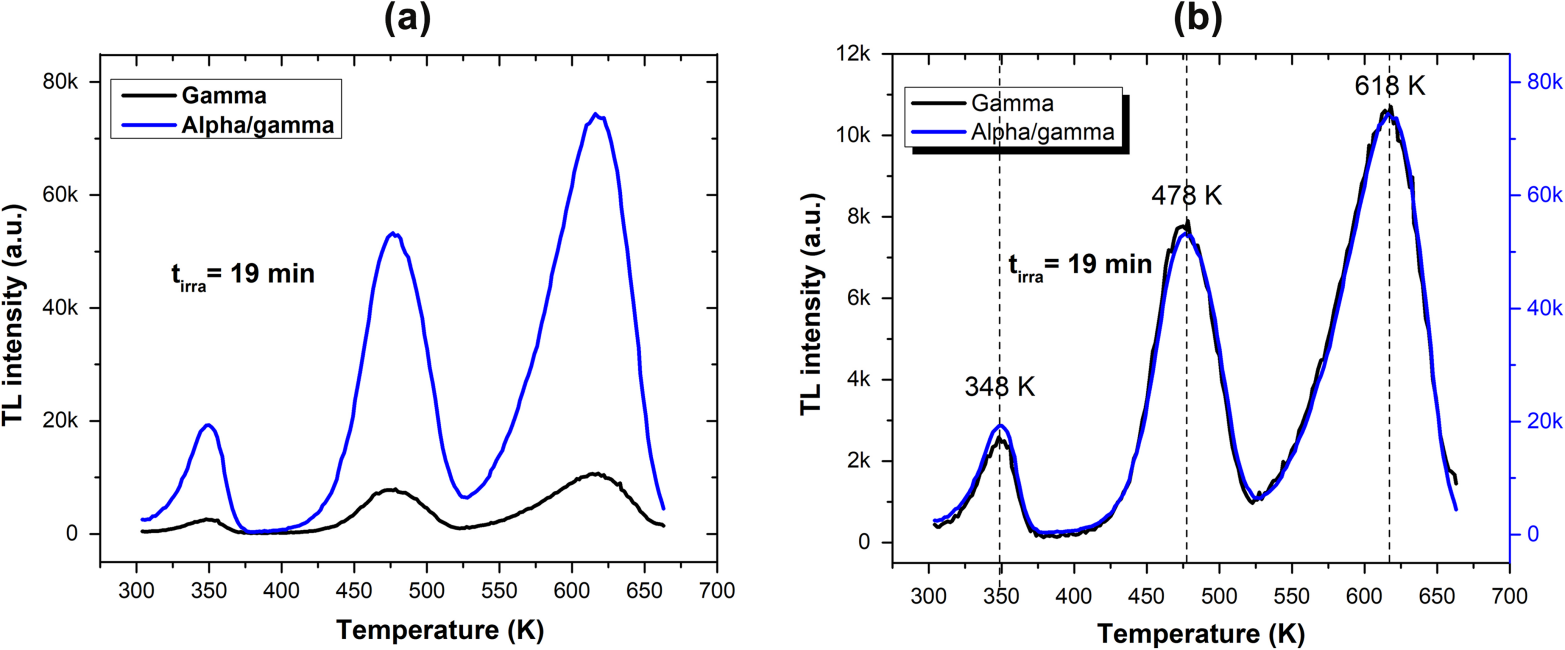
(a) TL glow curves for BeO irradiated with both types of radiation used in this study. (b) Comparison of the TL glow curves.

**FIGURE 2:**
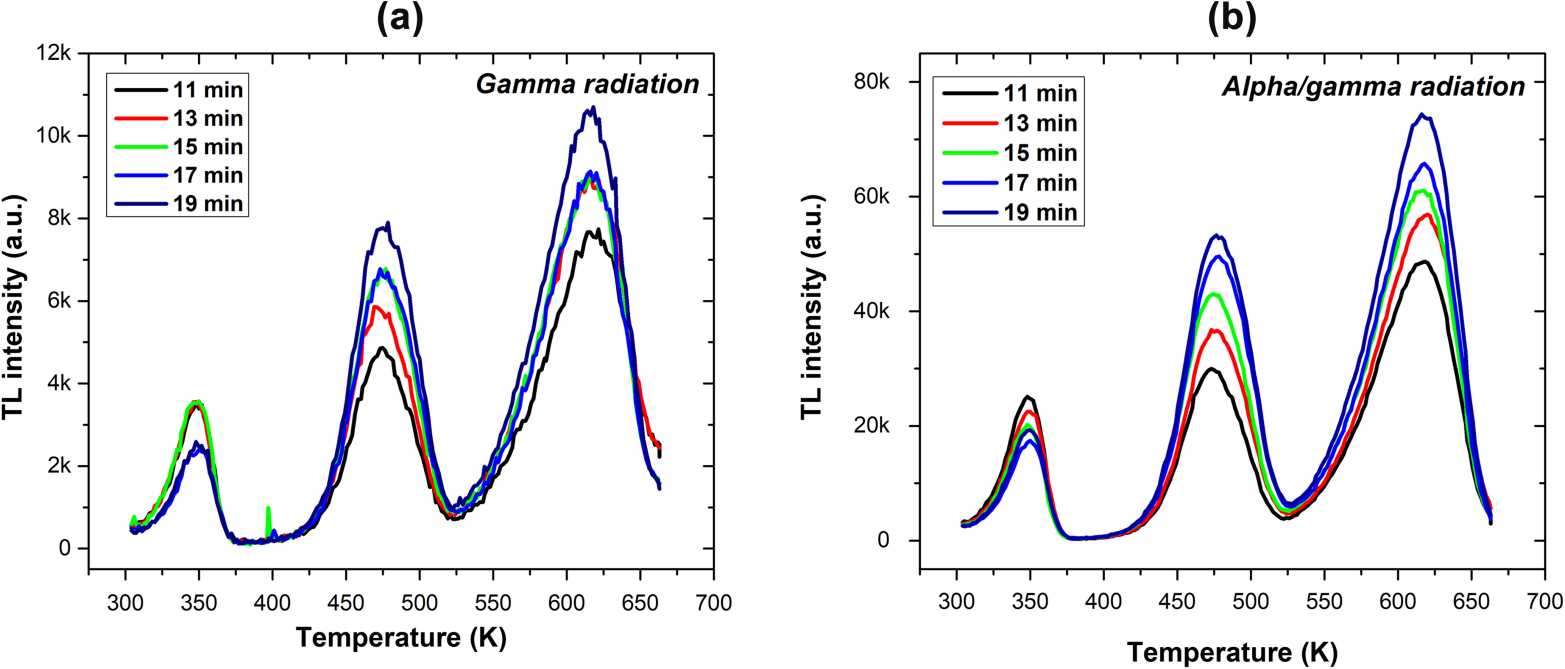
Evolution curves for (a) gamma and (b) alpha/gamma radiation.

Figure 1a shows the BeO glow curves obtained after 19 min for both gamma and alpha/gamma radiation, indicating that the TL glow curve for alpha/gamma radiation is approximately 85% more intense than that for gamma radiation, as can be inferred by calculating the total area under these curves. Figure 2b shows a comparison of the curves. Both curves are composed of three peaks centered at 348, 478, and 618 K, hereafter referred to as Peak 0, Peak 1, and Peak 2, respectively. This figure illustrates that, for both irradiations, the shape of the curves and the TL peak positions remain unchanged. This suggests first‐order kinetics for each peak, an observation that agrees with previous reports [3, 16].

Several research studies have investigated the thermoluminescent (TL) properties of Thermalox 995. These studies consistently report a predominant TL peak centered around 478 K, but they differ regarding the maximum temperature position of the second TL peak. For instance, Azorín‐Nieto et al. [16] and Martínez‐Baltezar and Azorín‐Nieto [3] both observed the second peak at 586 K. Although they employed the same gamma irradiation dose and identical readout parameters, Azorín‐Nieto et al. [16] used a Cs‐137 gamma source, whereas Martínez‐Baltezar and Azorín‐Nieto [3] used a Co‐60 source, which emits higher‐energy gamma rays (1.17 and 1.33 MeV, respectively).

In contrast, Algarve and Caldas [2] used X‐ray radiation (0.1 MeV), like the energies employed in the present study (0.060 MeV). Their results show that the position of the second TL peak agrees with that observed in this work, occurring at approximately 618 K. Additionally, Sasaki et al. [15] compared their findings with those of Martínez‐Baltezar and Azorín‐Nieto [3]. However, they employed very low heating rates (0.133, 0.05, and 0.005 K/s), which led to significant differences in the glow curve profiles compared with those reported by Martínez‐Baltezar and Azorín‐Nieto [3].

To enable a proper comparison, Sasaki et al. [15] used their activation energy and frequency factor values to estimate the TL peak temperature at a heating rate of 5 K/s. Their calculations predicted that, under these conditions, the peak position could be approximately 20 K higher than that reported by Martínez‐Baltezar and Azorín‐Nieto [3]. Sasaki et al. [15] suggested that this discrepancy might be related to differences in post‐annealing temperature. However, they also employed significantly higher‐energy electromagnetic radiation (6 MeV) and a light‐based heating system, both of which could contribute to the observed differences.

All these studies suggest that the position of the second TL peak in Thermalox 995, when exposed to electromagnetic radiation, may be influenced by the energy of the incident photons.

Figure 2a shows the BeO glow curves measured at different gamma irradiation times, indicating that Peaks 0 and 2 do not provide a distinguishable response to the variation in gamma irradiation time in the studied range. Figure 2b presents the glow curves obtained under varying alpha/gamma irradiation conditions, where Peak 0 shows a decreasing trend over the studied interval, while Peaks 1 and 2 exhibit an increasing response.

**FIGURE 3:**
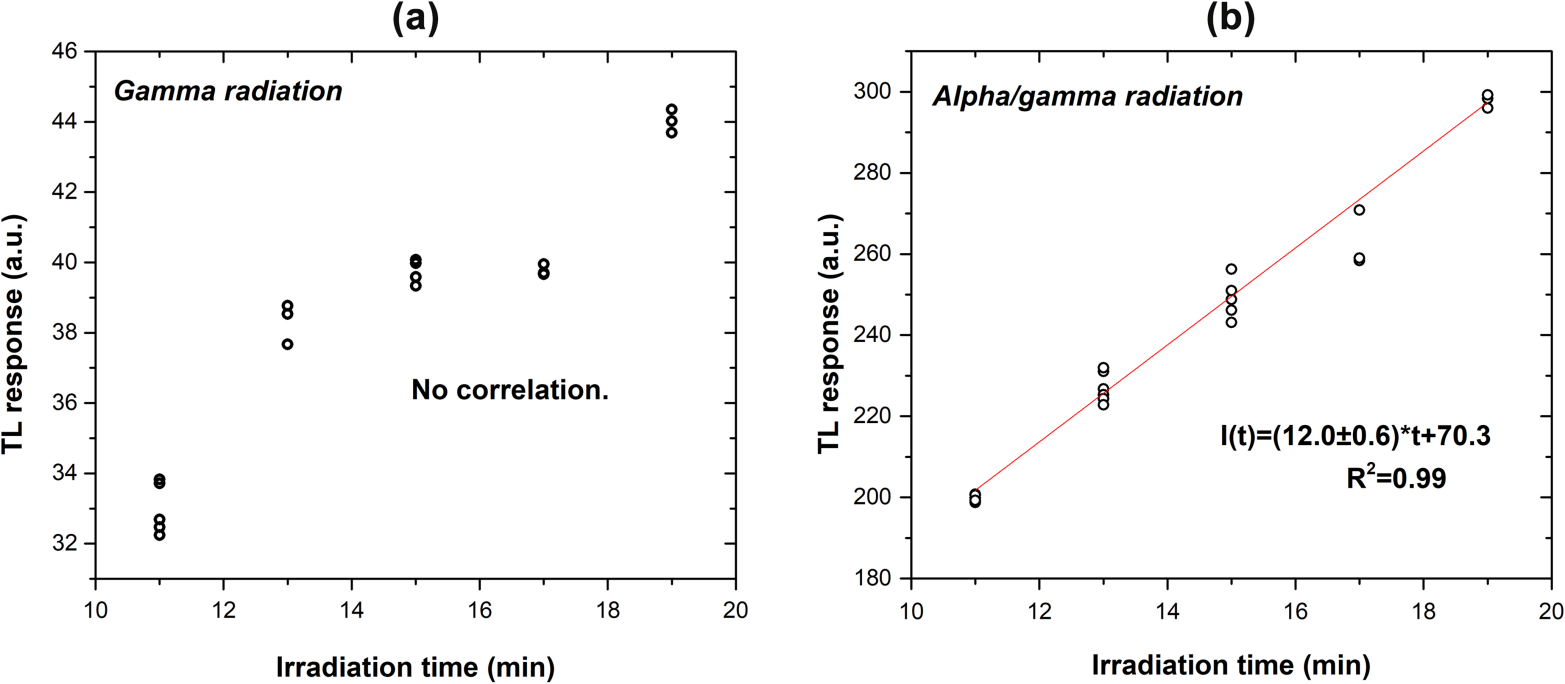
TL response as a function of irradiation time for whole TL curves for (a) gamma and (b) alpha/gamma radiation.

Figure 3a shows the TL response (total area) as a function of increasing gamma irradiation time, illustrating that there is no clear correlation between these two quantities. Figure 3b shows the TL response (total area) as a function of increasing alfa‐gamma irradiation, suggesting a linear correlation between these two quantities.

**FIGURE 4:**
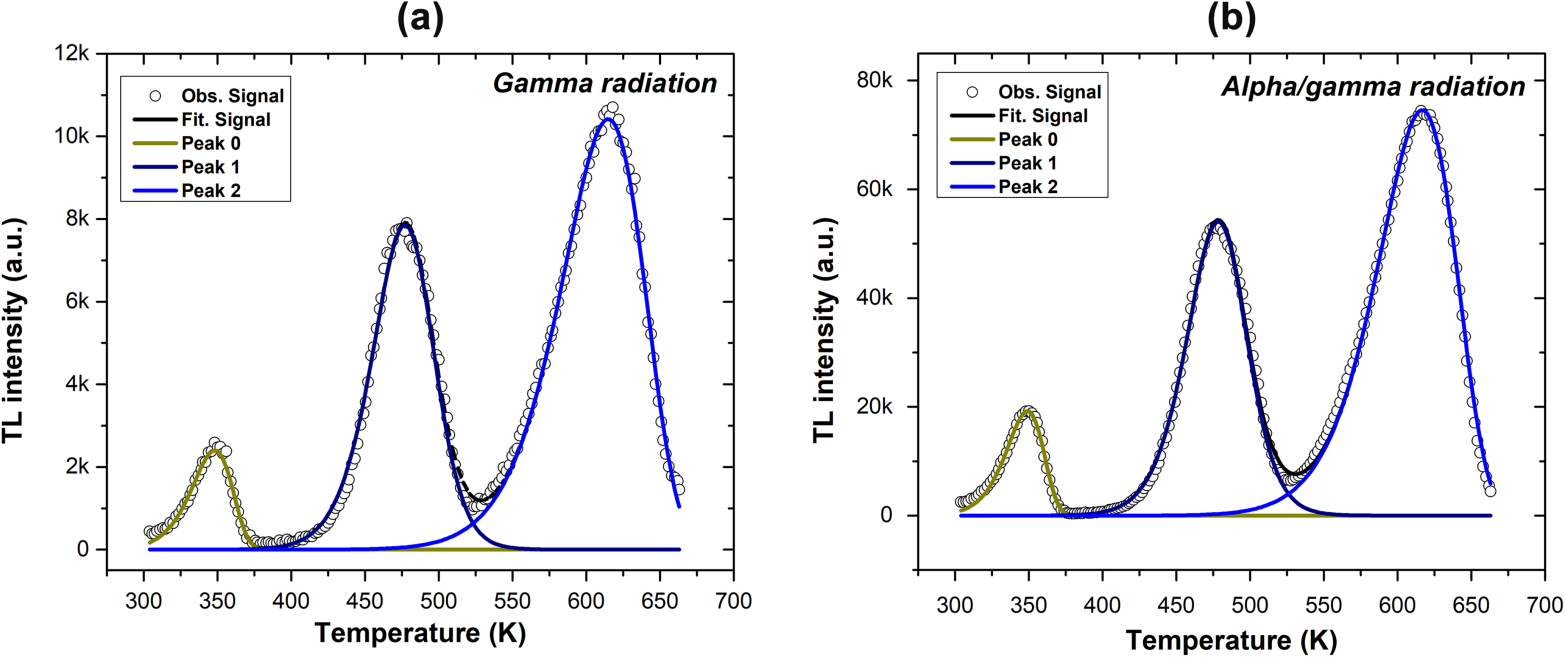
Deconvolution of TL glow curve for (a) gamma and (b) alpha/gamma radiation.

Figure 4a shows the results of the deconvolution method, which fits three peaks to the glow curve for 19 min of gamma irradiation at 348, 476, and 616 K. The FOM for the gamma irradiation fits ranges from 3 to 7. Figure 4b displays similar results, fitting essentially the same three components to the glow curve after 19 min of alpha/gamma irradiation. In this case, the FOM ranges from 2 to 5. Table 1 presents the values obtained for activation energy (E), temperature (K), kinetic order (b), and half‐life for each peak under both gamma and alpha–gamma irradiation (19 min), as determined by the deconvolution method.

**TABLE 1:** Kinetic parameters mean values obtained by deconvolution method for both types of radiation gamma (G) and alpha/gamma (A/G).

Parameter	Peak 0		Peak 1		Peak 2	
Radiation	G	A/G	G	A/G	G	A/G
Activation energy (eV)	0.76 ± 0.03	0.81 ± 0.02	1.18 ± 0.04	1.22 ± 0.03	1.11 ± 0.02	1.18 ± 0.01
Temperature (K)	348 ± 1	348 ± 1	476 ± 1	477 ± 2	616 ± 1	615 ± 2
Kinetic order	1.01 ± 0.01	1.01 ± 0.02	1.51 ± 0.10	1.57 ± 0.08	1.07 ± 0.05	1.01 ± 0.01
Half‐life	131 s	168 s	1.92 a	5.07 a	522 a	2067 a

The kinetic order for Peaks 0 and 2 is approximately 1.01 ± 0.05 for both irradiation types. For Peak 1, the kinetic order is close to 1.51 ± 0.10 and 1.57 ± 0.08 under gamma and alpha/gamma irradiation, respectively. Table 1 also shows that the activation energies of TL peaks induced by alpha/gamma radiation are higher than those induced by gamma radiation. There are no significant differences between the activation energies reported by Algarve and Caldas [2] (1.20 and 1.32 eV for the 1 and 2 peaks, respectively), Azorín‐Nieto et al. [16] (1.02 and 1.30 eV), Martínez‐Baltezar and Azorín‐Nieto [3] (1.10 eV for both peaks), and the present study (1.18 ± 0.04 and 1.11 ± 0.02 eV). However, these values differ significantly from those reported by Sasaki et al. [15] (1.15 and 1.74 eV). This discrepancy may be associated with either the high radiation energy used or the heating system employed by Sasaki et al.

Table 2 reports the maximum TL intensity values for each peak and for each type of radiation. The uncertainty in each measurement is included in Table 2. Peak 0 presents the maximum uncertainty values for both kinds of radiation used in this study. This is maybe caused by the high fading observed at this peak and reported in Table 3. Peaks 1 and 2 present uncertainty values equal or less than 2% for each kind of radiation used in this study, except for a 17 min irradiation time. These results suggest that BeO Thermalox 995 could have a good precision dose, which is a requirement for medical applications.

**TABLE 2:** Maximum intensity and corresponding uncertainty for each peak as a function of irradiation time.

	Peak 0		Peak 1		Peak 2	
Time (min)	G	A/G	G	A/G	G	A/G
11	3.34E+03 ± 12%	2.62E+04 ± 8%	4.80E+03 ± 2%	3.00E+04 ± 1%	7.45E+03 ± 1%	4.88E+04 ± 1%
13	3.47E+03 ± 13%	2.22E+04 ± 7%	5.87E+03 ± 2%	3.72E+04 ± 2%	8.75E+03 ± 2%	5.71E+04 ± 1%
15	3.91E+03 ± 10%	2.01E+04 ± 9%	6.76E+03 ± 1%	4.37E+04 ± 1%	9.11E+03 ± 3%	6.09E+04 ± 2%
17	2.33E+03 ± 9%	1.74E+04 ± 10%	6.83E+03 ± 6%	5.05E+04 ± 2%	8.94E+03 ± 5%	6.56E+04 ± 2%
19	2.45E+03 ± 10%	1.91E+04 ± 5%	7.98E+03 ± 1%	5.43E+04 ± 1%	1.04E+04 ± 2%	7.41E+04 ± 2%

**TABLE 3:** Comparison between experimental fading results and theoretical predictions for Peak 0.

Radiation	Theoretical	Experimental	Relative error
Sample 1	130 s	101 ± 5 s	28%
Sample 2	95 s	101 ± 8 s	6%
Sample 3	95 s	95 ± 6 s	1%
Sample 4	86 s	94 ± 5 s	9%
Sample 5	95 s	87 ± 5 s	8%

**FIGURE 5:**
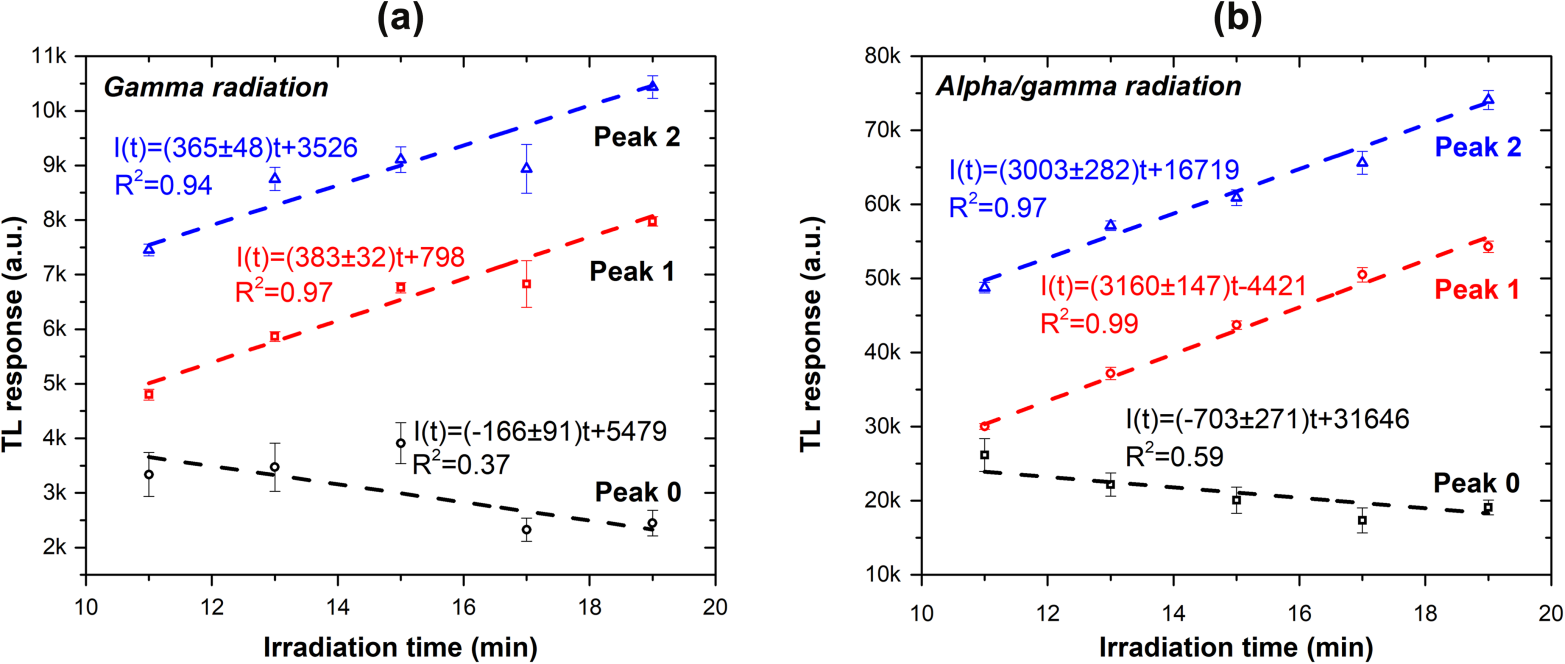
TL maximum intensity as a function of irradiation time for each glow peak for (a) gamma and (b) alpha/gamma radiation.

Figure 5a shows the maximum TL intensity as a function of gamma irradiation time for each glow peak, while Figure 5b presents the corresponding data for alpha/gamma irradiation. As shown in Figure 5a for gamma irradiation, Peaks 0 and 2 exhibit low *R* ^2^ values (0.37 and 0.94, respectively), whereas Peak 1 displays a higher and acceptable *R* ^2^ value of 0.97. In contrast, Figure 5b shows that under alpha/gamma irradiation, Peaks 1 and 2 exhibit more pronounced linear behavior, with higher *R* ^2^ values of 0.99 and 0.97, respectively, compared with those under gamma irradiation. Peak 0, however, shows saturation behavior and a decreasing trend as the alpha/gamma irradiation time increases.

**FIGURE 6:**
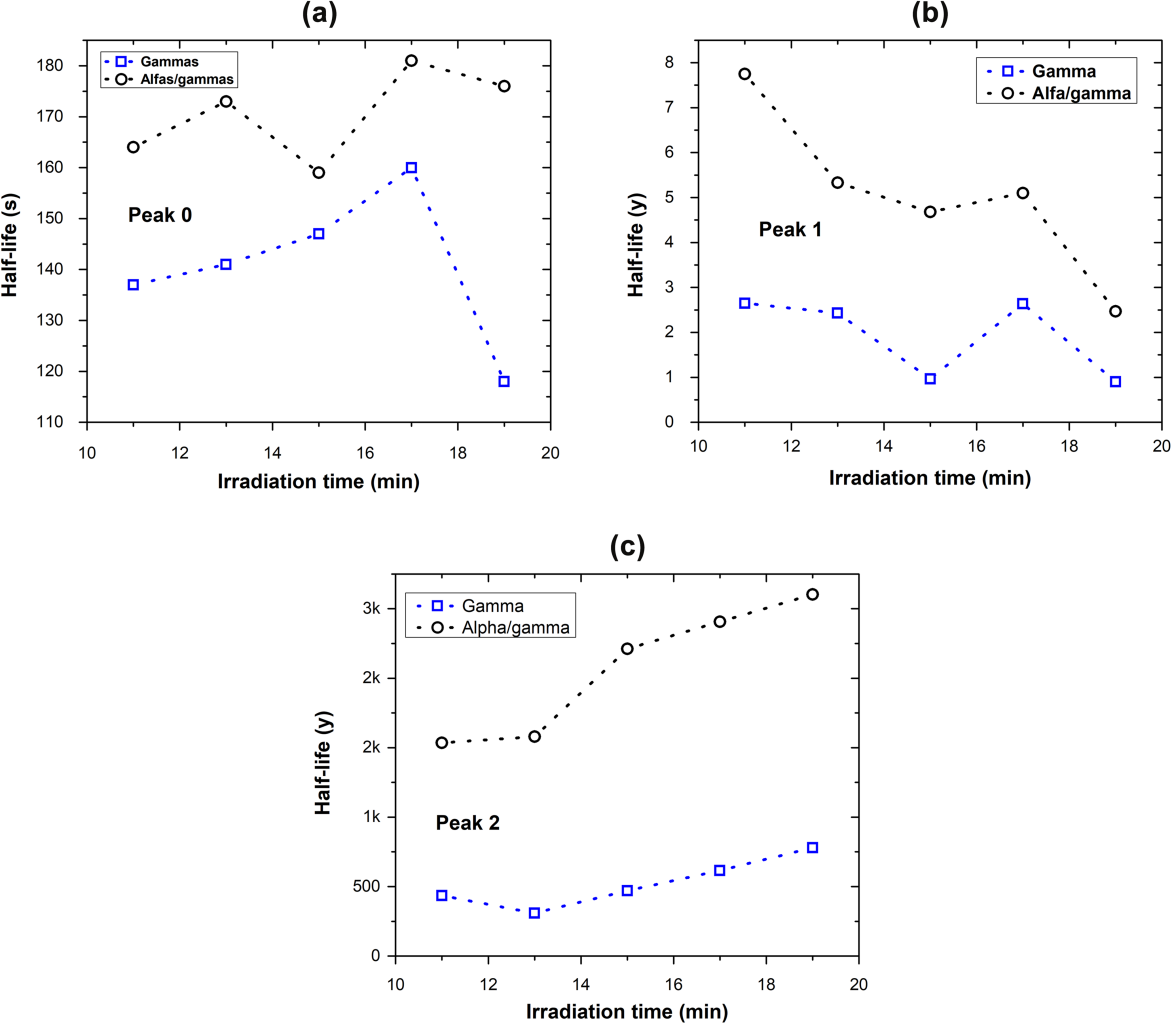
Half‐life as a function of irradiation time for each glow peak. (a) Peak 0, (b) Peak 1, and (c) Peak 2.

Figure 6 shows the half‐life of each peak as a function of irradiation time for both types of radiation used in this study. The main observation from this figure is that the half‐life values of the TL glow curve peaks under gamma irradiation are lower than those corresponding to the peaks under alpha/gamma irradiation. Figure 6b indicates that the half‐life of Peak 1 tends to decrease as the irradiation time of gamma or alpha/gamma radiation increases, with this tendency being more pronounced for alpha/gamma irradiation. In contrast, Figure 6c shows that the half‐life of Peak 2 tends to increase with increasing irradiation time of gamma or alpha/gamma radiation. These findings indicate that the trap‐filling mechanisms associated with each TL peak are distinct. Further studies are required to clarify the underlying processes.

In this study, the authors chose to use low gamma radiation doses (ranging from 0.45 to 4.3 mGy), whereas Azorín‐Nieto et al. [16] used a high gamma radiation dose of 15.6 Gy. Using the parameters reported by Azorín‐Nieto et al. [16] for a heating rate of 5 K/s, the half‐lives of the first (E = 1.02 eV, b = 1.1, Tm = 477 K) and second (E = 1.30 eV, b = 1.1, Tm = 586 K) TL peaks were calculated considering the storage temperature (298 K) used in this study. The resulting half‐lives are 0.26 years (approximately 95.8 days) and 6687 years for the first and second peaks, respectively. These results are consistent with the observed trend in which the first peak decreases with increasing radiation dose, while the second peak increases.

**FIGURE 7:**
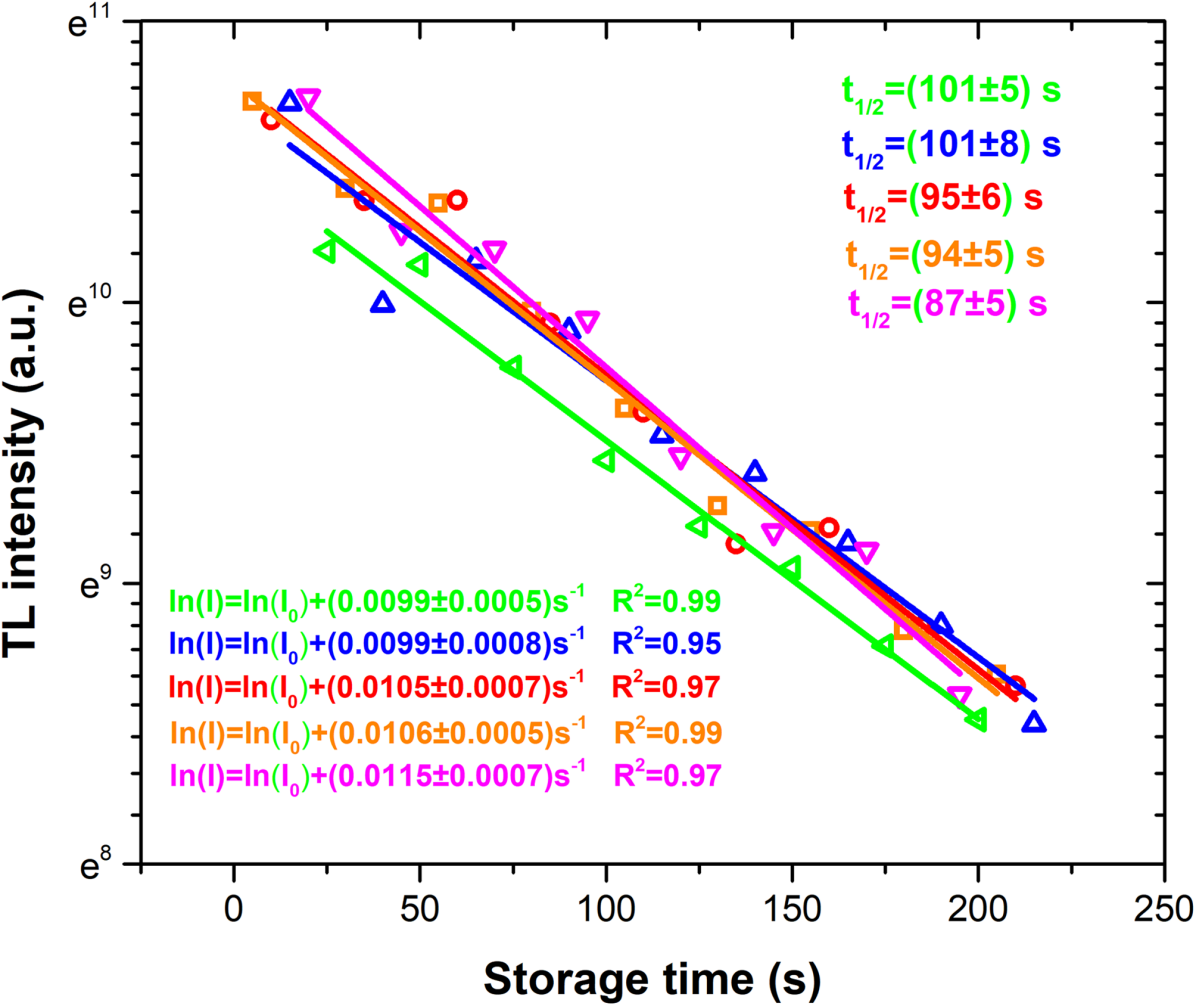
Semi‐logarithmic plots of the fading behavior for Peak 0 in five Thermalox 995 samples. The linear equations shown correspond to the best fit the experimental data. The half‐life for each sample was calculated as the reciprocal of the slope of the respective linear fit.

Figure 7 shows the results of the analysis of fading experiments for five samples; the results for these experiments agree with the results for the half‐life of the Peak 0 reported in Table 1. These experiments enable us to determine the discrepancy between experimental measurements and theoretical calculations as reported in Table 3.

## 4 Conclusions

In conclusion, the results show significant differences between the BeO TL glow curves induced by gamma radiation and those induced by alpha/gamma radiation. Although both BeO glow curves obtained in this work are composed of three peaks centered at 348, 478, and 618 K, the activation energy and half‐life for each peak are higher in the case of alpha/gamma radiation than for gamma radiation. For both types of radiation, the results also show that the half‐life values depend on the irradiation time. For the peak at 478 K, the half‐life tends to decrease as the gamma or alpha/gamma irradiation time increases, whereas for the peak at 618 K, it tends to increase with increasing irradiation time. This suggests that the mechanisms of the traps filling associated with each peak are different. Deconvolution results indicate that, for both gamma and alpha/gamma irradiation, the peak centered at 478 K exhibits the best linear irradiation time‐response behavior. Given that half‐life is a key parameter related to the stability of the TLD system, further investigations should be carried out on Thermalox 995 and other important dosimetric materials to examine the half‐life of individual glow peaks under different radiation sources.

## Conflicts of Interest

The manuscript titled “Calculation of the half‐life for the TL signal of BeO under gamma and alfa/gamma irradiation” has not been published previously and is not under consideration for publication elsewhere. Additionally, none of the authors have a conflict of interest to disclose.

## Data Availability

Research data are not shared.
